# Harnessing the Infectivity and Stability of Bacteriophage ΦNF‑1 in a Phage-Based Product for Sustainable Nitrification Control

**DOI:** 10.1021/acs.est.5c14179

**Published:** 2026-05-01

**Authors:** Gloria Vique, Pedro Blanco-Picazo, Aina Trenchs, María Dolores Ramos-Barbero, Pablo Quirós, Sergio Atares, Ignasi Salaet, Lorena Rodríguez-Rubio, Maite Muniesa, Laura Sala-Comorera

**Affiliations:** † Department de Genètica, Microbiologia i Estadística, 16724 Universitat de Barcelona, Diagonal 643, Annex, Floor 0, 08028 Barcelona, Spain; ‡ Departamento de I+D+i de Fertinagro Biotech S.L, Polígono Industrial La Paz, Berlín, parcela 185, 44195 Teruel, Spain; § Institut d’Investigació en Nutrició i Seguretat Alimentària (INSA), Universitat de Barcelona, Recinte Torribera, Av. Prat de la Riba 171, Edifici La Masia, 08921 Santa Coloma de Gramenet, Spain

**Keywords:** bacteriophages, inhibitor, nitrification, Nitrosomonas, photoprotector, persistence, biocontrol

## Abstract

The intensive use of nitrogen fertilizers sustains crop productivity but causes substantial nitrogen losses, greenhouse gas emissions, and water contamination through nitrification-driven leaching and volatilization. Although chemical nitrification inhibitors reduce these effects, concerns over persistence, toxicity, and impacts on nontarget organisms limit their sustainability. As a biological alternative, bacteriophages can selectively suppress ammonia-oxidizing bacteria (AOB) responsible for nitrification. This study examines the virulent bacteriophage ΦNF-1, the first known phage infecting soil AOB of the genus *Nitrosomonas*. ΦNF-1 reduced nitrification in fertilized soils inoculated with *Nitrosomonas europaea* and, to a lesser extent, in fertilized soils containing native AOB communities. Beyond its previously reported hosts, ΦNF-1 also propagated in *Nitrosomonas oligotropha* and *Nitrosospira multiformis*, expanding its known host range. ΦNF-1 remained infectious for over six months in aqueous suspensions at 4–20 °C and neutral to alkaline pH, but declined rapidly under acidic conditions. In agricultural soils, infectivity persisted for up to six months, though recovery was lower in sandy soils. A calcium carbonate-based photoprotectant significantly enhanced phage survival under UV and sunlight exposure. Overall, ΦNF-1 shows strong potential as a stable, host-specific biological nitrification inhibitor to improve nitrogen use efficiency and reduce environmental impacts of fertilization.

## Graphical abstract

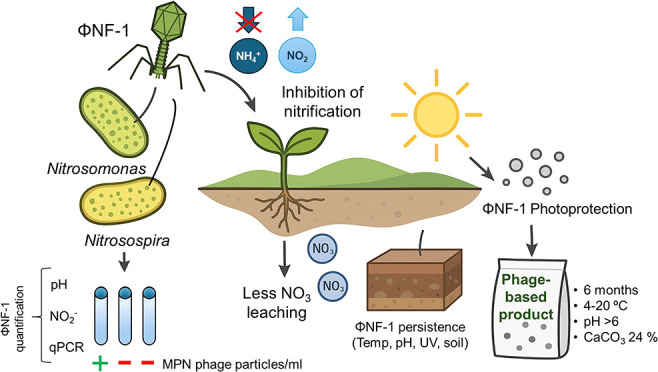

## 1 Introduction

Modern agriculture is highly dependent on nitrogen (N) fertilizers, such as ammonium or urea, to sustain and increase crop productivity.^1,2^ However, a significant fraction of applied N is lost to the environment through nitrification, a microbially driven process. In soils, ammonia (NH_3_) is sequentially oxidized by specialized microorganisms: first to nitrite (NO_2_^–^) by ammonia-oxidizing bacteria (AOB) and archaea (AOA), and then to nitrate (NO_3_^–^) by nitrite-oxidizing bacteria (NOB).^1,3,4^ The most common AOB belong to the genera *Nitrosomonas*, *Nitrosospira*, and *Nitrosococcus*, while AOA are often represented by *Nitrosophaera* and *Nitrosopumilus* ^5,6^ Among NOB, *Nitrobacter* and *Nitrosococcus* are well-known representatives.^7,8^ Complete ammonia oxidizers (comammox), such as certain *Nitrospira* species, can perform both nitrification steps within a single organism.^4,9^ In most cases, however, nitrification depends on a mutualistic symbiosis between ammonia and nitrite oxidizers. Besides soils, nitrifying bacteria are abundant in ecosystems with high ammonia availability, such as wastewater and rivers, lakes, and streams receiving large inputs of organic matter.^6,10,11^

Nitrification is a crucial step in the global biogeochemical N cycle, and soils act as major regulators of N fluxes.^11^ In parallel, denitrification reduces NO_3_^–^ through successive intermediates, NO_2_^–^, nitric oxide (NO), and nitrous oxide (N_2_O), to produce dinitrogen (N_2_).^12^ Additionally, mineral ammonium can be converted to gaseous NH_3_ and lost to the atmosphere.^1^

Despite their widespread use, N-based fertilizers are often inefficient, with up to 70% of applied N lost from agricultural soils.^13^ Losses occur mainly through NH_3_ volatilization and emissions of N_2_O, a potent greenhouse gas.^1,14^ In N-enriched soils, the activity of AOB, AOA, and NOB intensifies, reducing N availability to crops. To compensate, farmers apply larger amounts of fertilizer, but excess N is prone to leaching into water bodies, causing contamination^15,16^ or volatilization, with harmful effects on ecosystems. Ammonia oxidation is the rate-limiting step of nitrification and thus determines how quickly ammonium is converted to NO_3_^–^, causing many of the associated environmental problems in agricultural settings.^16^

To improve fertilizer efficiency by retaining N in the ammonium form, chemical nitrification inhibitors have been widely adopted. These compounds generally work by blocking the active site of the ammonia monooxygenase enzyme^2^ and are applied by blending with fertilizers or spreading directly onto the soil.^2,15^ However, despite over five decades of research, the efficacy of chemical nitrification inhibitors in mitigating N_2_O emissions is typically short-lived, lasting only a few weeks. Furthermore, there are concerns about their environmental fate; residues can accumulate in ecosystems, enter food chains, and contaminate water systems, and their ecotoxicological effects on nontarget microorganisms, plants, animals and humans remain incompletely understood.^17,18^

Given these limitations, bacteriophages have emerged as a promising biocontrol alternative. Phage infection leads to host cell lysis and death, a mechanism that has been successfully applied to combat phytopathogenic bacteria in agriculture,^19,20^ pathogens in veterinary and clinical settings, and foodborne bacteria in the food industry.^21–23^ Recently, the virulent phage ΦNF-1 was isolated and proposed as the first phage specifically targeting AOB for nitrification inhibition.^24^ This phage is reported to infect at least three *Nitrosomonas* species (*Nitrosomonas europaea*, *Nitrosomonas communis* and *Nitrosomonas nitrosa*), has a 41.5 kb genome and has the characteristics of virulent phages; lack of integrase genes, toxins, and antibiotic resistance genes. Its closest known relative (a phage infecting *Sphaerotilus*) shares only 60.7% nucleotide identity, suggesting that ΦNF-1 may represent a distinct genus. In vitro assays confirmed its ability to inhibit AOB growth, obtaining a 2 log_10_ units increase in ΦNF-1 particles.^24^ This was accompanied by reduced ammonium depletion in the phage-treated cultures, thereby lowering the nitrification rate.^24^

Although ΦNF-1 has been proposed as a novel biocontrol tool for inhibiting nitrification, it is essential to assess its persistence and infectivity under diverse environmental conditions before incorporating it into a fertilizer formulation. We therefore assessed the stability of ΦNF-1 suspensions across a range of temperatures and pH values, as well as in different soil types under laboratory and greenhouse conditions. We also tested the use of photoprotective agents as a strategy to enhance phage persistence under sunlight, thereby improving its potential effectiveness in the field.

## 2 Materials and Methods

### 2.1 Bacteriophage, Bacterial Strains and Media

The phage ΦNF-1 (GenBank accession number OL634959) was used to infect the following AOB species: *N. europaea* (ATCC 25978), *Nitrosospira eutropha* C91,^25^ *Nitrosomonas oligotropha* Nm45,^26^ *Nitrosomonas ureae* Nm10,^26^ *Nitrosospira multiformis* ATCC 25196,^27^ and *Nitrosospira briensis* C-128.^28^ Bacteria were grown in DSMZ 1583 medium supplemented with 1 mL of a trace element solution (DSMZ https://www.dsmz.de/microorganisms/medium/pdf/DSMZ_Medium1583.pdf), omitting cresol red solution.

### 2.2 Bacterial Growth

Nitrifying bacteria are slow-growing microorganisms and do not generate sufficient bacterial mass to cause quantifiable turbidity in the liquid culture medium for spectrophotometric monitoring. Moreover, as these bacteria do not produce confluent lawns on agar, preventing the formation of plaques of lysis by the phages, the evaluation of phage propagation cannot be done by double agar layer, the most widely used technique for phage enumeration.^29^ Therefore, bacterial growth was monitored by measuring the pH reduction of the culture media using pH indicator test papers (Fisher Scientific). Cultures were incubated in the dark at 28 °C for 15 days and the pH values were recorded daily.

### 2.3 Measurement of Nitrification Activity

Nitrification kinetics in AOB was quantified to determine bacterial activity by measuring NO_2_^–^ accumulation in the medium using the colorimetric assay described by Hink.^30^ Briefly, a standard series was prepared in the first row of a 96-well plate containing 0–200 μM NO_2_^–^ diluted in 0.1 M KCl. In each well, 100 μL of sample was mixed with 60 μL of diazotizing reagent 1 (sulfanilamide solution containing 5.0 g sulfanilamide in 100 mL of 3.3 M HCl). Subsequently, 20 μL of coupling reagent 2 (NED solution containing 0.30 g N-(1-naphthyl) ethylenediamine dihydrochloride in 100 mL of 0.12 M HCl) was added. Absorbance was measured at 540 nm using a photometer (Fisher Scientific) to determine the NO_2_^–^ concentration.

#### 2.3.1 Nitrification Inhibition by ΦNF-1 in Soil

NO_2_^–^ was analyzed in fertilized agricultural soil (soil 1) using a microcosm experiment. Four hundred grams of soil were placed in two-liter polypropylene containers and fertilized with 150 kg N ha^–1^ as solid (NH_4_)_2_SO_4_. The containers were irrigated to reach water-holding capacity (≈32%) and subsequently watered twice weekly, with the amount adjusted according to weight loss. One day after fertilization, *N. europaea* culture was added to the corresponding soil samples to a final concentration of 10^6^ CFU g^–1^. Eight days after bacterial inoculation, a dialyzed ΦNF-1 phage suspension was added to achieve an estimated final concentration of 10^6^ particles g^–1^ and 10^8^ CFU g^–1^ of bacterial cells (MOI 0.01), and samples were incubated for 10 days in the dark at 28 °C. The results from soils containing *N. europaea* were compared in the presence and absence of phage. The experiment was performed in three independent replicates.

In addition, fertilized soil without *N. europaea* inoculation was assayed in the absence and presence of ΦNF-1 to evaluate the role of the phage in inhibiting nitrification in native AOB populations.

Mineral N was extracted from soil samples with 0.1 M KCl using a 1:5 (w/v) soil-to-extractant ratio. The extracts were used to measure NO_2_^–^ by a colorimetric method, as described above, at at 0, 1, 2, 5, 8, and 10 days. The propagation of ΦNF-1 and the abundance of its *Nitrosomonas* host were evaluated at the same times by qPCR as described below.

### 2.4 Standard qPCR Procedures

The propagation of ΦNF-1 in the cultures was evaluated by qPCR. DNA was extracted using the QIAamp DNA Blood Mini Kit (Qiagen GmbH), following the manufacturer’s instructions, and suspended in a final volume of 200 μL sterile bidistilled water. qPCR assays were run on the StepOne Real Time PCR System (Life Technologies) in 20 μL reactions. Standards with known DNA concentration were prepared from gBlocks Gene Fragments and were used for efficiency calculation and as positive controls.

A ΦNF-1-specific TaqMan assay included TaqMan Environmental Master Mix 2.0 (Life Technologies). Relative quantification was performed using the primers described for ΦNF-1 (UP: 5′-GCAGTTAGTTCTGATCCGTGGAA-3′, LP: 5′-ATAAGCCCTATGATCGGGTAAGG-3′ and TaqMan probe: 6FAM-TTAGTTGGTAGGGAGACATA- MGBNFQ).^24^ The ΦNF-1 qPCR assay showed a linearity (*R* ^2^) of 0.998 and an amplification efficiency of 99.2%.

Bacterial DNA was quantified using Power SYBR Green (Life Technologies) in a one-step reaction following the manufacturer’s protocol, with primers targeting the *amoA* gene (UP: 5′-TGGCAGGTGACTGGGATTTC-3′ and LP: 5′-CCACCAAAGGTACGCAGTGA-3′).^24^ According to genetic databases, this assay targets the *amoA* gene in *N. europaea* and many other *Nitrosomonas* species and is also capable of detecting the gene in a few *Nitrosospira* species, including *N. multiformis* and *N. briensis*. Each qPCR run included positive and negative controls. Relative quantification was calculated using the comparative threshold cycle (*C_t_*) method,^31^ with *C_t_* values reported as the mean of three independent experiments. The identity of the amplicons was confirmed by the melting curve analysis. The *amoA* qPCR assay showed *R* ^2^ value and efficiency of 0.995 and 101.9%, respectively.

### 2.5 Viral Stock Preparation

Phage ΦNF-1 was propagated on exponentially growing *N. europaea* (ATCC 25978) at a multiplicity of infection (MOI) of 0.01 for 48 h. The culture medium was then adjusted to pH 8, and phage lysate was filtered through a 0.22 μm low-protein-binding poly­(ether sulfone) filter (Millex-GP). Viral particles were concentrated using Vivaflow 30 kDa cassettes (Sartorius) and dialyzed against distilled water to remove N compounds (ammonium and NO_2_^–^) until a final volume of 20 mL was reached.

### 2.6 Infectivity Assays

Infectivity of dialyzed ΦNF-1 suspensions was assessed against exponential-phase cultures of *N. europaea*, *N. eutropha*, *N. oligotropha*, *N. ureae*, *N. multiformis* and *N. briensis*. Assays were conducted at a MOI of 0.01. Cultures and phage-free controls were incubated for 48 h in the dark at 28 °C. Before infection, pH of the cultures was adjusted to pH 8.0. Propagation was monitored daily for 8 days postinoculation by measuring: (i) pH reduction using pH indicator test papers, (ii) NO_2_^–^ concentration in the media (as per Section 2.3), and (iii) viral particle abundance by qPCR. A strain was considered susceptible if a significant decrease in NO_2_^–^ and a corresponding increase in viral DNA were observed compared to the control. All experiments were carried out in triplicate.

### 2.7 Quantification of Infectious Particles

As phage ΦNF-1 does not form plaques of lysis (owing to the inability of host strains to produce confluent lawns on agar), the number of infectious particles in phage suspensions was determined using a Most Probable Number (MPN) assay. At each time point, suspensions were serially diluted in a phage buffer (7 g Na_2_HPO_4_, 1.5 g KH_2_PO_4_, and 5 g NaCl, supplemented poststerilization with 10 mL of 0.1 M MgSO_4_ and 10 mL of 0.01 M CaCl_2_).^32^ Each dilution was used to infect 2 mL of an exponential-phase host culture, in triplicate (Figure 1).

**Figure 1:**
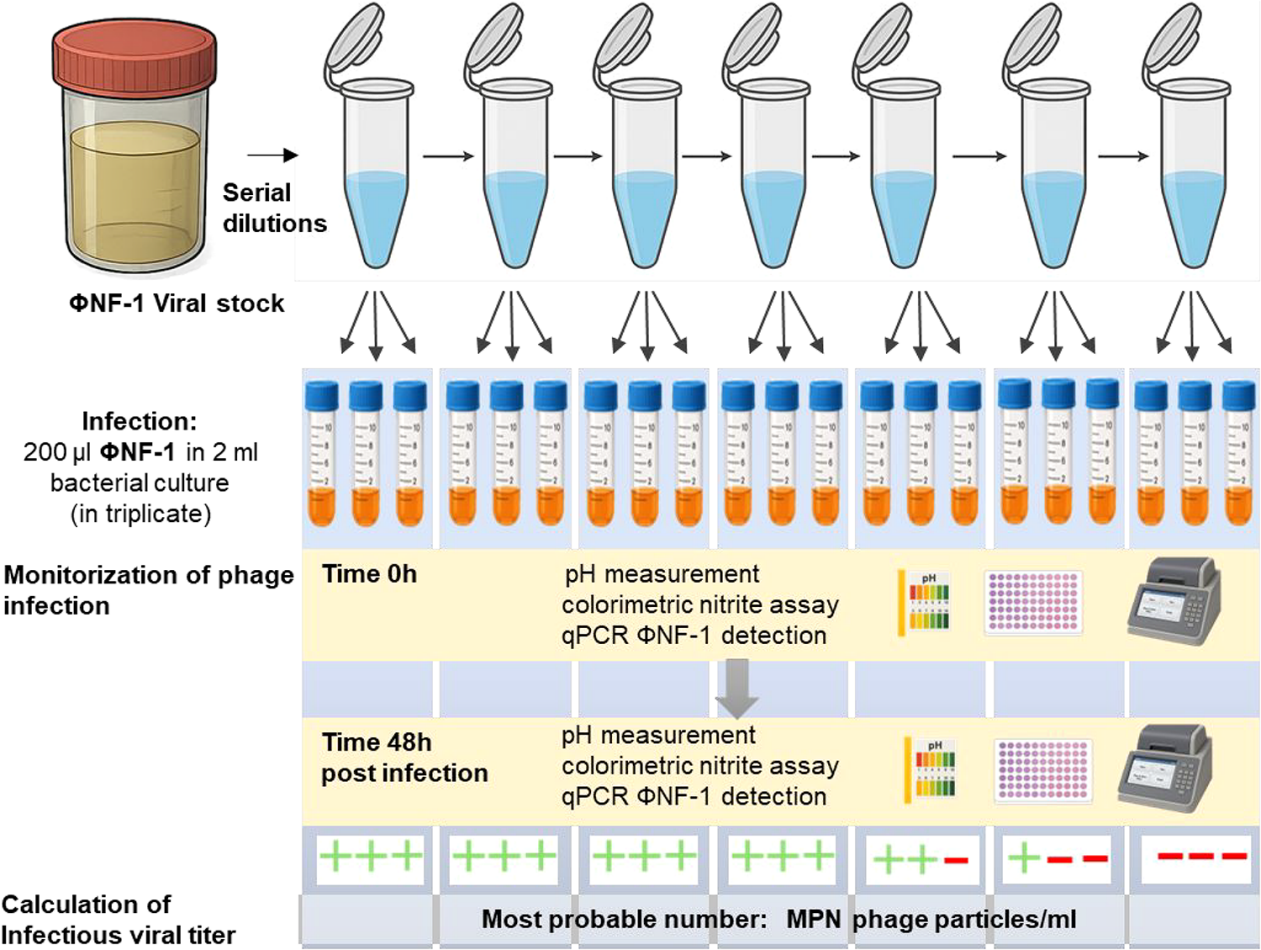
Workflow for the quantification of infectious phage ΦNF-1 particles based on the MPN. This procedure shows the steps to evaluate infectious particles at each time taken when the suspension was evaluated at the different conditions.

To assess phage propagation, aliquots were collected immediately after inoculation (time 0) and at 48 h of incubation for the measurement of pH, NO_2_^–^ concentration, and ΦNF-1 *C_t_* values. Samples were scored as positive for phage infection if the ΦNF-1 *C_t_* value after 48 h decreased by ≥3 cycles compared to time 0, indicating successful replication. Samples with a change in *C_t_* value of <3 were scored as negative. Based on the distribution of positive/negative outcomes across dilutions, infectious phage titers (MPN phage particles/ml) were calculated using the ISO MPN Calculator (http://standards.iso.org/iso/7218/).

### 2.8 Stability Assays of ΦNF-1 at Different Temperatures and pH

A stock of ΦNF-1 (7.8–8.7 log_10_ MPN/mL), dialyzed against distilled water using a 30 kDa Vivaflow cassette (Sartorius), was divided into 20 mL aliquots and stored under different conditions. Thermal stability was assessed at 4 °C, 15 °C, 20 °C, and 30 °C. pH stability was tested at pH 3, 5, 6, 7, and 8 by adjusting with HCl 37% (pH 3–7) or 10% KHCO_3_ (pH 8). All suspensions were incubated in darkness and pH-adjusted suspensions were stored at 4 °C. The titer of infectious particles was determined immediately (day 0) and on days 1, 7, 14, and 21, and then monthly for up to 6 months. At each time point, titers were quantified by infecting an *N. europaea* culture and confirming phage propagation by qPCR (Figure 1). DNA was extracted from viral suspensions using the QIAamp DNA Blood Mini Kit (Qiagen GmbH) and its integrity was assessed by qPCR.

To investigate if phage reduction at low pH (3 and 5) was due to aggregation, an additional experiment was performed. After 24 h of incubation at pH 3 and 5, suspensions were neutralized to pH 7 by adding 1N NaOH, agitated for 1 h at 4 °C, and the infectious phages were quantified as described above.

### 2.9 Recovery of ΦNF-1 in Soils

Phage recovery was assessed in four soil types: two agricultural soils, one commercial garden soil, and one sandy soil. Ten grams of sterilized soil were spiked with 1 mL of concentrated ΦNF-1 suspension (final concentration 8.08 log_10_ MPN/g) and homogenized on a shaker for 10 min. Spiked soils were incubated in triplicate at 20 °C in the dark. Phages were extracted from soil after 24 h.^32^ Each 10 g soil sample was mixed at a 1:2 (W/V) ratio with a buffer (10% beef extract powder, pH 8.5; Fisher) and homogenized using a wrist-action shaker (900 osc/min) for 10 min at room temperature. The homogenate was centrifuged at 3000*g* for 15 min and the supernatant was filtered through a 0.22 μm poly­(ether sulfone) membrane (Millipore). To calculate phage recovery, filtrates were serially diluted, used to infect cultures, and infectious titers were determined by the MPN method detailed in Section 2.7 (Figure 1). Recovery efficiency was calculated as the logarithmic reduction of phages recovered from the soil relative to the theoretical spiked concentration.

### 2.10 Stability of ΦNF-1 in Soils

The persistence of ΦNF-1 was evaluated in two agricultural soils. Soils were previously sterilized and divided into 10-g portions for each sampling time point. Each portion was spiked with ΦNF-1 to a final concentration of 7.04–7.38 log_10_ MPN/g and thoroughly mixed. All samples were incubated in triplicate at 20 °C in the dark. Infectious phage particles were enumerated weekly for the first month and monthly thereafter for up to six months. Phage elution from the soil was performed as described in Section 2.9. The resulting filtrate was serially diluted, and the infectious titer was determined using the MPN method (Section 2.7, and Figure 1).

### 2.11 Persistence of ϕNF-1 under UV and Sunlight with Photoprotective Agents In Vitro and in Soil

The efficacy of two commercial products as photoprotectants for ΦNF-1 was tested: Amino acid 22% (Fertinagro Biotech), a levorotatory amino acid–based fertilizer, and Vegepron Sun (UPL), a calcium carbonate-based foliar fertilizer. Amino acid 22% acts on development, flowering, and promotes the recovery of weakened crops, it stimulates root development and soil microbiota, increasing soil fertility, and it unlocks trace elements in the soil, facilitating their assimilation. Amino acid 22% is applied either by foliar spraying or directly to the soil through irrigation water. CaCO_3_ in Vegepron Sun amends calcium deficiencies, enhances the plant’s mechanical resistance to abiotic factors, acting as both an osmoprotectant and a shield against sun exposure thanks to the opacity of the product.

Two concentrations (12% and 24%) of each photoprotectant were prepared with distilled water. Phage suspensions were diluted 1:10 into these solutions or into water (untreated control) to obtain final concentrations of 6.54–8.01 log_10_ MPN/mL and mixed by shaking. Samples in uncovered sterile plastic Petri dishes (Figure 2A) were placed 10 cm beneath a germicidal UV lamp (8 W, 253.7 nm; model G30T8, Sankyo Denki) prewarmed for 15–30 min. Aliquots were collected at 0, 0.5, 1, 2, 3, 4, 6, 8, or 24 h of exposure for enumeration of infectious phages depending on the experiment. The UV fluence rate was calculated using the equation *D* = *I* × *T*, where *D* is the dose, *I* is the intensity (0.099 mW/cm^2^), and *T* is exposure time (seconds). The fluence rate was calculated to be 0 (0 h), 178.2 (0.5 h), 356.4 (1 h), 712.8 (2 h), 1069.2 (3 h), 1425.6 (4 h), 2138.4 (6 h), 2851.2 (8 h), and 8553.6 0 (24 h), mJ/cm^2^. As a control, phage suspensions without photoprotectant were incubated in the same conditions. All were tested in triplicate.

**Figure 2:**
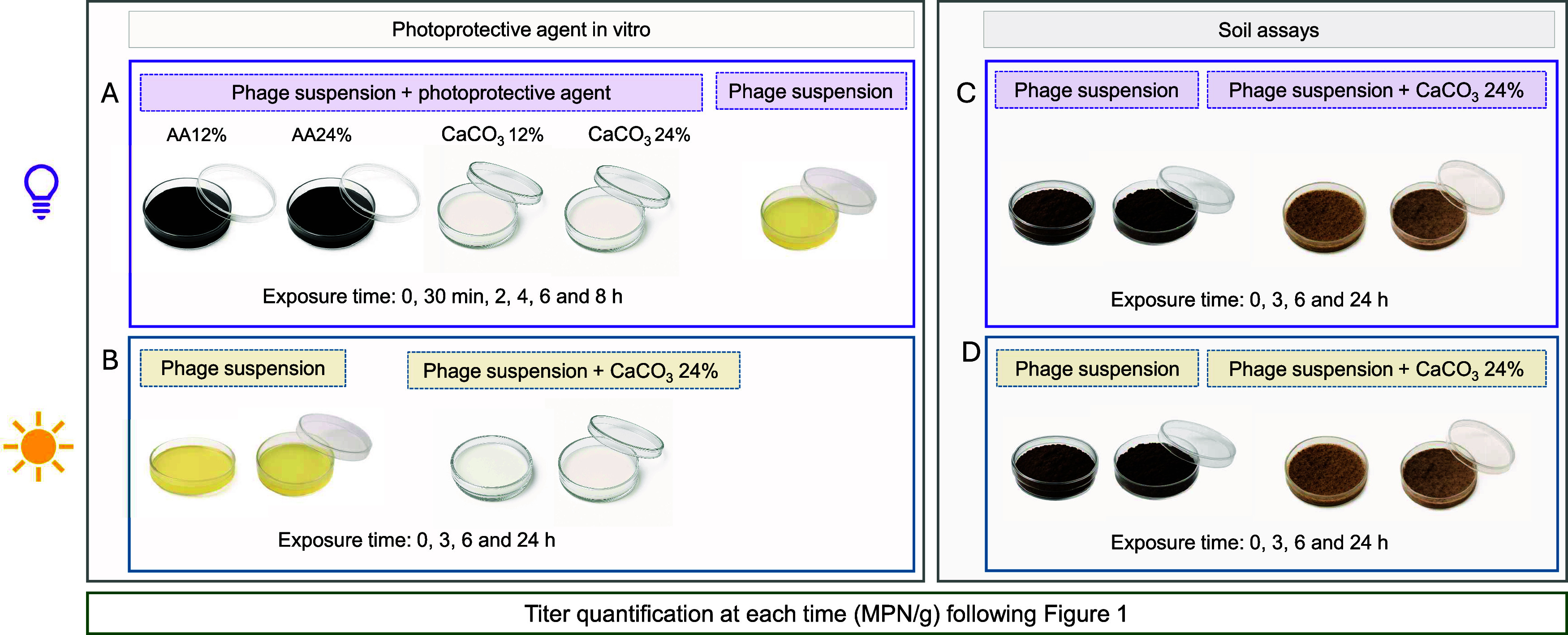
Experimental design for evaluating ΦNF-1 persistence under UV light and natural sunlight, with and without photoprotective agent. Infectious phage particles were quantified by the MPN method as described in Figure 1. (A) Phage suspensions exposed to UV light for 8 h in the presence of two photoprotectors (Amino acid 22% and CaCO_3_ at 12 and 24%). (B) Phage suspensions with 24% CaCO_3_ exposed to natural sunlight for 24 h and compared to the suspensions without photoprotectant in covered or uncovered Petri dishes. (C) Phage suspensions, with or without 24% CaCO_3_, mixed with agricultural soil and exposed to UV light for up to 24h in covered or uncovered Petri dishes. (D) Same as (C), but under natural sunlight.

In the natural sunlight assays (Figure 2B), only CaCO_3_ at 24% was tested, as it provided the best protection under UV light (see Section 3). Phage suspensions were prepared as above and phage-protectant mixtures were placed in plastic Petri dishes, either uncovered or covered to simulate greenhouse conditions. The infectious titers were determined at 0, 3, 6, and 24 h after exposure outdoors. Sunlight intensity (W/m^2^) was obtained from the Catalan Forecast Service (*Servei Meterorològic de Catalunya*, *Spain*).

Aliquots of 10 g of sterilized agricultural soil were spiked with 1 mL of phage suspension and 9 mL of CaCO_3_ (24%), resulting in a final ΦNF-1 concentration of 5.79 log_10_ MPN/g soil. Control samples contained phages without a photoprotectant. Soils were homogenized with a wrist-action shaker (900 osc/min, 10 min, room temperature) and transferred to Petri dishes. Eight dishes were exposed to the 8-W UV lamp (Figure 2C) and eight were exposed to natural sunlight outdoors (Figure 2D); in both cases, four were covered to simulate greenhouse conditions. Infectious titers were measured at 0, 3, 6, and 24 h. All experiments were performed in triplicate.

### 2.12 Electron Microscopy Studies

Ten μL of cultures of the different host strains infected with the phage suspension were dropped onto copper grids with carbon-coated Formvar films, negatively stained with 2% ammonium molybdate (pH 6.8) for 30 s and examined under a Tecnai G2 Spirit TWIN transmission electron microscope (FEI Technologies Inc. Oregon, US) operating at 120 kV.

### 2.13 Data Analysis

Statistical analysis and data plotting were performed using GraphPad Prism10 (GraphPad Software). Normality of the data of nitrification in soil was assessed using Kolmogorov–Smirnov normality test. At the end of the experiment, the mean of NO_2_^–^ concentration of soils treated with bacteriophages and without bacteriophages were compared using unpaired *t* test with a significance value of *p* < 0.05.

The decay rates of infectious phage and genomic DNA were modeled using a first-order decay model proposed by Chick,^33^ a one-phase nonlinear decay model, and a segmental linear model.

The first-order decay model was defined as

$$C_{t}=C_{0}\times e^{-\mathrm{kt}}$$

The one-phase nonlinear model was defined as

$$C_{t}=\left(C_{0}-\mathrm{Plateau}\right)e^{-\mathrm{kt}}+\mathrm{Plateau}$$

where *C*_f_ and *C*_0_ are the concentrations of infectious phages (MPN/mL or MPN/g) at time *t* and time 0, respectively, and *k* is the decay rate constant.

The segmental linear model^33^ was defined as

$$C_{t}=C_{f0}e^{-k_{f}t}+C_{s0}e^{-k_{s}t}$$

where *k*_f_ corresponds to the fast decay rate and *k*_s_ to the slow decay rate. *C*_f0_ and *C*_s0_ are the initial concentration in the fast and slow decay period, respectively.

The goodness of fit for each model, *r* ^2^ and root-mean-square error (RMSE) were calculated. The best fitting model was chosen based on the Akaike information criterion or the extra sum-of-squares F-test. The time required for a 90% reduction in the initial concentration (*T*_90_) was calculated by solving the equation of the best fitting model considering *C_t_* equal to one.

## 3 Results

### 3.1 Inhibition of Nitrification by Phage ϕNF-1

Nitrification activity in the soil incubations was estimated from the net production of NO_2_^–^ in the soil extracts over time. This approach is based on NO_2_^–^ and assumes that nitrification causes microbial oxidation to NO_2_^–^.

In soils inoculated with *N. europaea*, higher NO_2_^–^ production was observed in the phage-free control compared with samples treated with ΦNF-1 at a MOI of 0.01 (Figure 3A). After evaluating a range of MOIs (1, 0.1, 0.01, and 0.001; Figure S1) phage propagation was confirmed at all tested MOIs except at MOI 0.001. MOI of 0.01 was identified as optimal for nitrification inhibition as it showed performance comparable to higher MOIs while requiring fewer phages (Figure S1). Differences are significant (*p* < 0.05) after day 5 and the maximum inhibitory effect is of 49.5% at day 10. Linear production rates in the presence of phage over the early linear phase (0–10 h) are of 317.0 μM/day, whereas in the absence of phage is of 755.6 μM. (Figure 3A), indicating strong suppression of nitrification activity. qPCR analysis of ΦNF-1 in these samples showed phage propagation (negative Δ*C_t_*) over time (Figure 3C) indicative of infection and phage propagation. As a result, the concentration of the inoculated *N. europaea* decreased. This decline became statistically significant (*p* < 0.05) from day 5 onward when comparing the *N. europaea* culture exposed to phage with the bacterial control lacking phage, and the difference continued to increase over time (Figure 3C).

**Figure 3:**
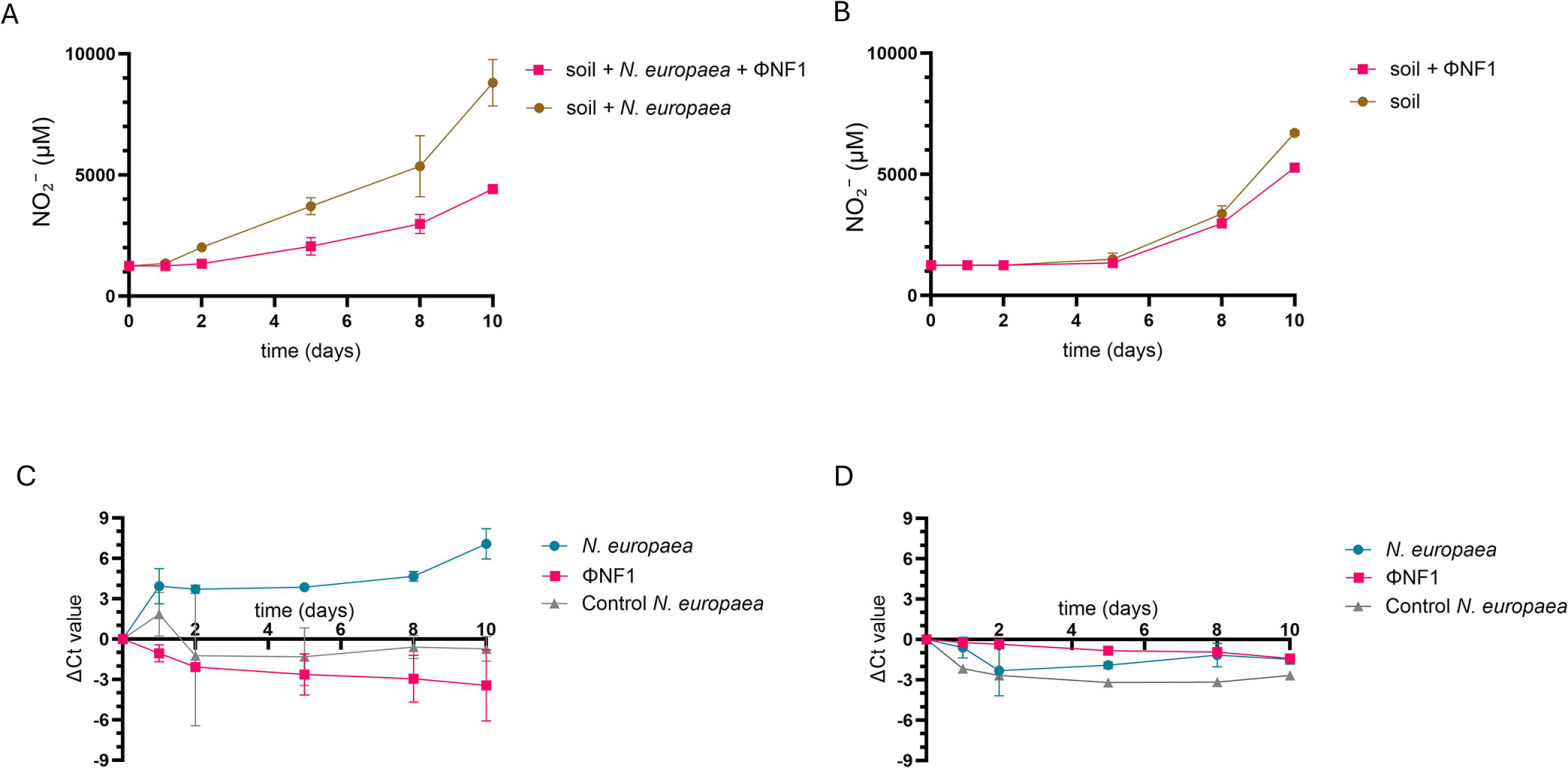
Inhibition of nitrification by phage ΦNF-1 in soil. Inhibition of nitrification measurements was based on NO_2_^–^ production in fertilized soil microcosms, phage propagation (ΦNF-1) was monitored by the reduction of *C_t_* values in the cultures, bacterial infection (lysis) was monitored by the increase of *C_t_* values in the cultures in comparison to the noninfected strain (control *N. europaea*). (A) NO_2_^–^ measurements (μM), in fertilized soil microcosms inoculated with *N. europaea* in the absence or presence of phage ΦNF-1. (B) NO_2_^–^ measurements in fertilized soil microcosms with naturally occurring AOB populations in the absence or presence of phage ΦNF-1. (C) Phage propagation (ΦNF-1) and bacterial abundance (*N. europaea*) of experiments in (A) monitored by qPCR (Δ*C_t_* values) in the presence or absence (control) of ΦNF-1. (D) Phage propagation and bacterial abundance of experiments in (B) monitored by qPCR (Δ*C_t_* values) in the presence or absence (control) of ΦNF-1. Results are the average of three independent experiments. Error bars indicate standard deviation.

NO_2_^–^ accumulation in fertilized soil lacking *N. europaea* again showed moderately higher values in the samples without ΦNF-1 (Figure 3B), although the difference reached statistical significance (*p* < 0.05) only after 10 days of incubation. In the absence of ΦNF-1, NO_2_^–^ reached 6760 μM over 10 days, indicating active microbial nitrification. In contrast, soils inoculated with ΦNF-1 exhibited a slower rate of NO_2_^–^ accumulation, reaching 5360 μM over 10 days (Figure 3B), suggesting that the phage partially suppressed nitrifying microbial activity, plausibly by infecting only those natural AOB species susceptible to phage infection. These results in natural soil indicate a specific action of ΦNF-1 on *N. europaea* and related susceptible AOB, rather than a general soil toxicity or abiotic inhibition.

In accordance with NO_2_^–^ accumulation, qPCR assays showed a discrete yet consistent increase in ΦNF-1 abundance over time (Figure 3D), suggesting the phage may propagate in some natural AOB in the soil. Regarding the bacterial community, although no depletion comparable to that observed when *N. europaea* was artificially inoculated occurred, in the phage-treated sample there is still a lower detectable AOB abundance than in the noninoculated control (Figure 3D). According to the genomes available in the databases, the qPCR assay targeted *amoA* genes from several *Nitrosomonas* species and some, but not all, *Nitrosospira* species that may naturally be present in the soil. Thus, the reduced bacterial counts in the presence of ΦNF-1 was attributed to phage-susceptible AOB, whereas phage-insensitive species continued to grow, masking a more pronounced decline.

### 3.2 Host Range of ϕNF-1

Phage ΦNF-1 was previously shown to infect three *Nitrosomonas* species: *N. europaea*, *N. nitrosa* and *N. communis*.^24^ As other AOB are also common in agricultural soils and a phage able to infect more than one species would be more useful for our purposes, we wanted to extend the host range of the phage by testing additional potential hosts available in our collection. For this reason, the phage was used to infect cultures of *Nitrosomonas* (*N. eutropha*, *N. oligotropha*, and *N. ureae*) and *Nitrosospira* (*N. multiformis* and *N. briensis*). Phage propagation and the reduction of bacterial cell numbers were evaluated by monitoring qPCR *C_t_* values, changes in pH, and nitrification activity via NO_2_^–^ accumulation.

ΦNF-1 successfully propagated in *N. oligotropha* and *N. multiformis*, as evidenced by a decrease in phage *C_t_* values and a ∼1-log_10_ increase in phage gene copies within 8 days (Figure 4A,D). This replication was accompanied by inhibited bacterial growth, consistent with cell lysis. Additionally, bacterial *C_t_* values for both strains increased, NO_2_^–^ production was suppressed (remaining stable in infected cultures, while controls accumulated up to 10,000 μM NO_2_^–^ over the same period) (Figure 4B–E), and pH was constant in infected cultures but decreased to pH 6.5 in uninfected controls (Figure 4C–F), attributed to the bacterial growth.

**Figure 4:**
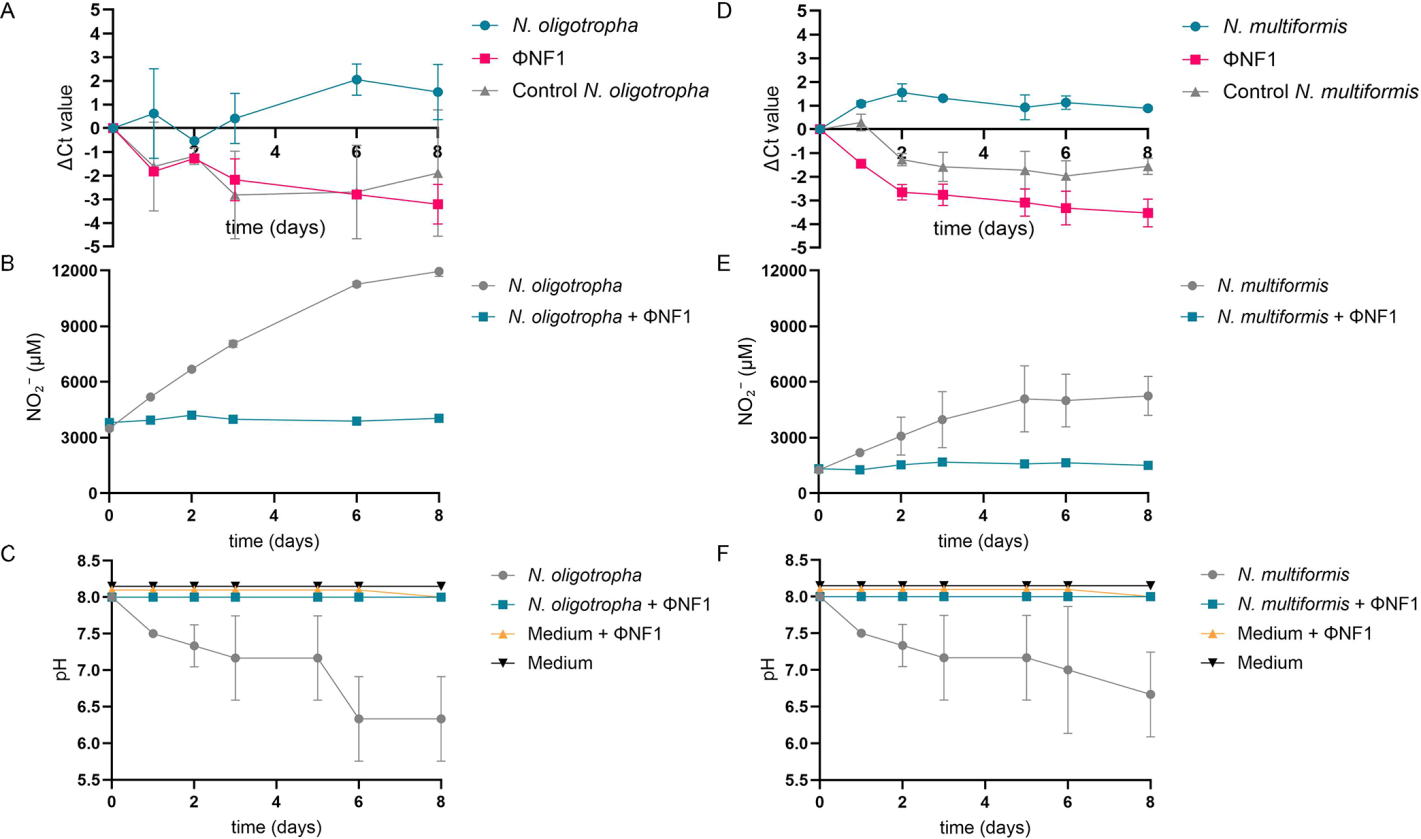
Infectivity dynamics of phage ΦNF-1 in *N. oligotropha* and *N. multiformis*. (A, D) Phage propagation and bacterial abundance were monitored by qPCR (Δ*C_t_* values) in the presence or absence (control) of ΦNF-1. (B, E) Nitrification activity was assessed by NO_2_^–^ accumulation, and (C, F) bacterial growth was monitored by pH variation. Data represent the mean of three independent experiments; error bars indicate standard deviation.

In contrast, no phage replication was detected in *N. eutropha*, *N. ureae*, or *N. briensis*. In these strains, phage gene copy numbers remained constant (data not shown), and bacterial growth proceeded unimpaired, with NO_2_^–^ accumulation and media acidification similar to those of uninfected controls (Figure S2).

These results expand the known host range of ΦNF-1. In addition to the three previously identified *Nitrosomonas* hosts,^24^ ΦNF-1 infects *N. oligotropha* and a member of the *Nitrosospira* genus, *N. multiformis*.

To verify that phage particles attach specifically to susceptible strains, cultures of the different bacteria were infected with phage ΦNF-1 at a MOI of 0.01 and incubated for 24 h in the dark at 28 °C. Infected cultures were gently centrifuged, and the pellets were examined by transmission electron microscopy. The results confirmed that susceptible strains (*N. europaea*, *N. nitrosa*, *N. communis*, *N. oligotropha*, and *N. multiformis*) displayed phage attachment (Figure 5A–E), whereas no phage particles were observed attached to the nonsusceptible strains (*N. eutropha*, *N. ureae*, or *N. briensis*) (Figure 5F–H). These findings confirm that ΦNF-1 binds specifically to the cell surface of susceptible bacterial strains, supporting previous observations.

**Figure 5:**
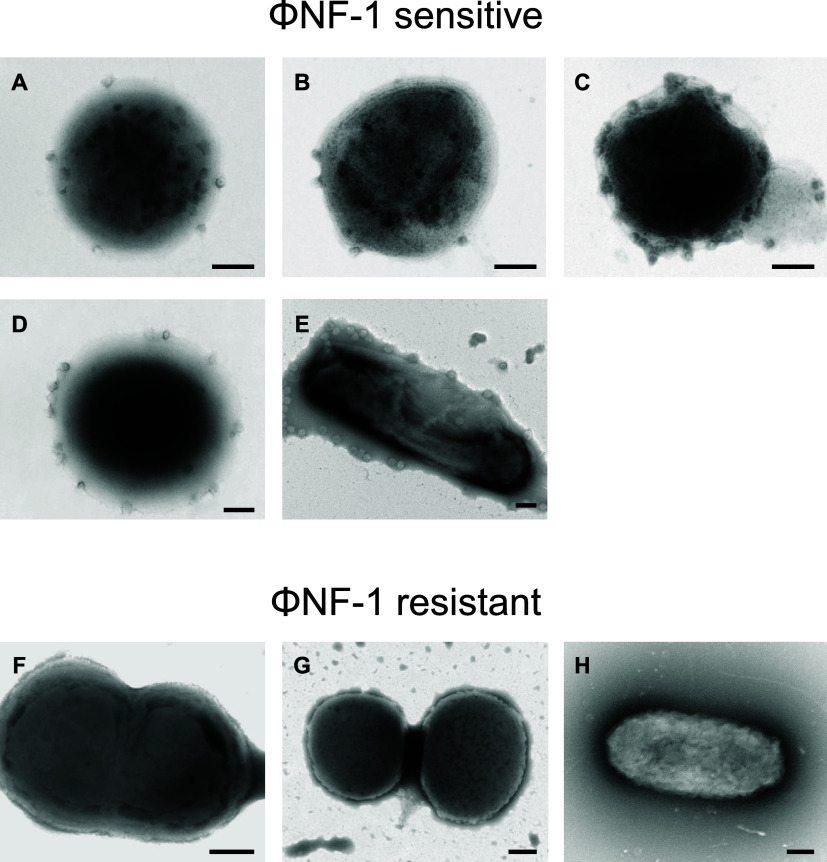
Electron micrographs of AOB cultures infected with ΦNF-1. Strains sensitive to the phage (A–E) and strains resistant to phage infection (F–H). (A) ΦNF-infected culture of *N. europaea*, (B) *N. nitrosa*, (C) *N. communis*, (D) *N.oligotropha*, (E) *N. multiformis*, (F) *N. eutropha*, (G) *N. ureae*, and (H) *N. briensis*. Bar = 200 nm.

### 3.3 Stability of ΦNF-1 Suspensions at Different Temperatures and pH

Before formulating fertilizer products, it was essential to assess the stability of infectious phage particles under various storage conditions, including temperature, pH, and long periods of storage. We therefore monitored the infectivity of ΦNF-1 particles in suspensions preserved in distilled water and incubated under different conditions in darkness for six months. As *Nitrosomonas* hosts do not form confluent lawns on semisolid agar^34^ phage titers could not be determined by plaque assays, making it difficult the proper validation of phage infectivity and propagation. Instead, the number of infectious phages in the phage suspension was quantified at each condition and time of assay by infecting nine host strain cultures with 10, 1, and 0.1 mL of the phage suspension (three replicates of each volume). Culture tubes showing infectious phages were determined by the variation of phage genomes by qPCR *C_t_* values compared between 0 and 48 h, and by nitrite assay. Those cultures showing and increase of phage genomes after 48 h but absence of NO_2_^–^-accumulation were considered positive for the presence of infectious phages, and the number of infectious phages was estimated using a MPN assay (Figure 1). Initial phage titers ranged from 7.8 to 8.7 log_10_ MPN/mL. Results are expressed as the logarithmic ratio of final to initial concentrations to facilitate cross-data set comparison.

ΦNF-1 particles remained relatively stable at the different temperatures over six months, showing a 1-log_10_ reduction only after 5 months. Decay followed a linear profile (Figure 6 and Table S1). At 4 °C, 15 and 20 °C, decay rates were similar (0.175–0.194 month^–1^), but slightly increased at 30 °C (0.246 month^–1^). At this higher temperature, the time required for a 1-log_10_ reduction (*T*_90_) was 4.05 months, compared with 5.14–5.71 months at lower temperatures.

**Figure 6:**
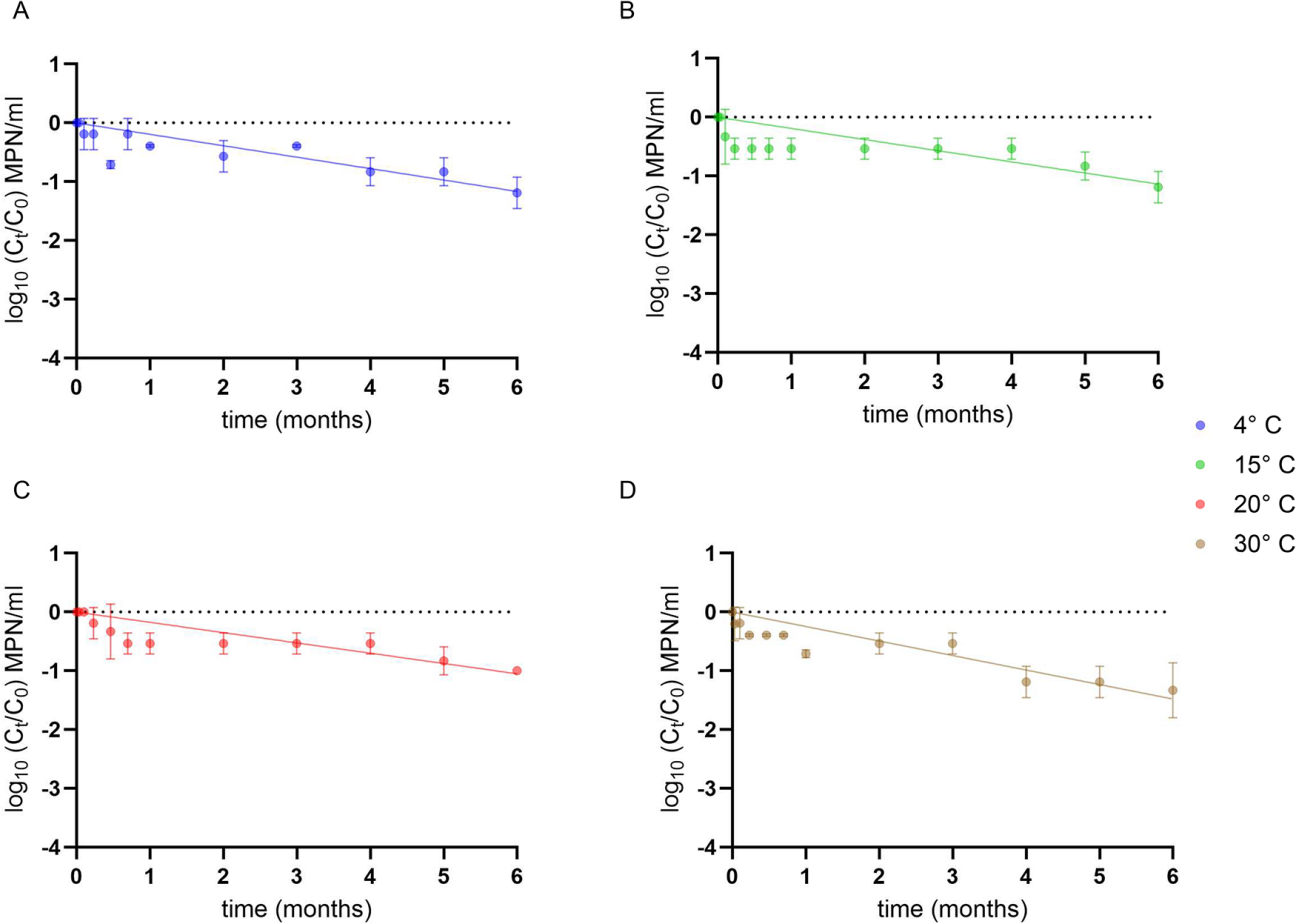
Persistence of phage ΦNF-1 at different temperatures. Decay curves of ΦNF-1 suspensions stored at 4 °C,15 °C, 20 °C, and 30 °C over 6 months. Error bars indicate standard deviation of three independent experiments.

Furthermore, considering the generation of a phage-based product, we tested different pHs of the media and analyzed the persistence of infectious phages simulating storage of the product for six months. We observed how phage stability varied across pH conditions. Complete and irreversible inactivation occurred within 24 h at pH 3 and 5 (Figure 7A), with infectivity not restored upon neutralization. At pH 6, decay followed a nonlinear one-phase model (Figure 7A), with a rate (*k* = 1.973 months^–1^) ∼10-fold higher than at pH 7 or 8. Infectious titers declined by 1 log_10_ within the first month (*T*_90_ = 0.48 months) before stabilizing. In contrast, decay at neutral and basic pH was linear and more gradual. The decay rate at pH 7 (*k* = 0.219 months^–1^) was faster than at pH 8 (*k* = 0.194 months^–1^), corresponding to *T*_90_ values of 4.5 and 5 months, respectively (Figure 7B,C). All regression parameters are provided in Table S1.

**Figure 7:**
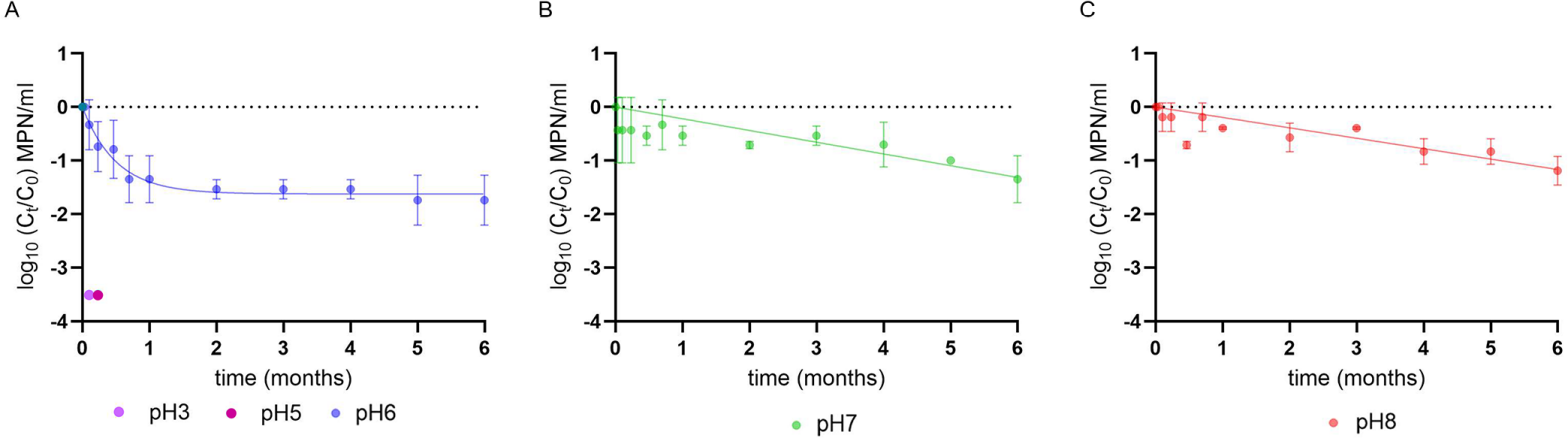
Persistence of phage ΦNF-1 at different pH values. Decay curves of phage ΦNF-1 stored at pH 3, 5, 6, 7, and 8 over 6 months. Error bars indicate standard deviation of three independent experiments.

Despite these losses in infectivity, phage genomic DNA remained relatively stable under most conditions (Figure S3). Long-term storage at all tested temperatures and pH values generally resulted in <1-log_10_ reduction in DNA copies over six months, except at pH 3 and 5, where rapid degradation occurred within the first 24 h.

### 3.4 Recovery of ΦNF-1 from Different Soils

Phage infectivity in soil can be affected by nonspecific adsorption to mineral particles, which depends on soil composition and pH. Before assessing the persistence of ΦNF-1 in soils, we first evaluated phage recovery after inoculation into different soil matrices. The inoculated phage load decreased by 0.7 and 1.2 log_10_ units in agricultural soils 1 and 2, respectively, and by 0.8 log_10_ units in commercial garden soil (Table S2). The greatest reduction occurred in sand, with a 1.7 log_10_ unit decrease (Table S2). These results confirm that soil properties, particularly particle size and composition, influence phage recovery, most likely through adherence or inactivation.

### 3.5 Stability of ΦNF-1 in Different Soils

To assess the potential efficacy of a ΦNF-1-based fertilizer, we evaluated the long-term stability of infectious phage particles in soil, as this determines both the duration of AOB infection and the required timing and frequency of application. Concentrated ΦNF-1 suspensions were inoculated into two agricultural soils at final concentrations of 7.04–7.38 log_10_ MPN/g and incubated in the dark. At each sampling point, 10 g of soil was resuspended in phage buffer, and infectious phage particles were determined using the MPN method. The phage concentrations and decay profiles were similar in both soils (Figure 8).

**Figure 8:**
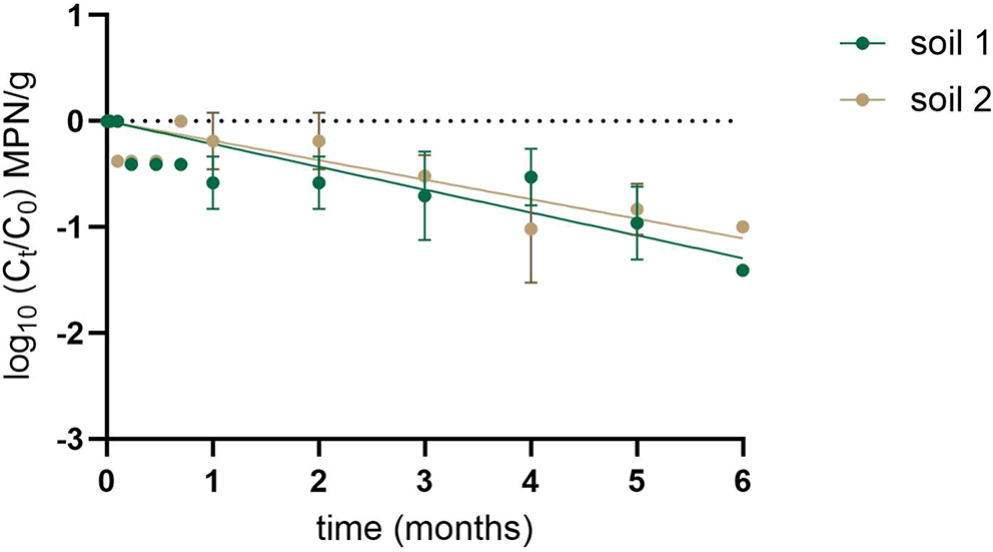
Stability of phage ΦNF-1 in agricultural soils. ΦNF-1 suspensions were spiked into agricultural soils and stored in the dark at room temperature for 6 months. Error bars indicate standard deviation of three independent experiments.

ΦNF-1 remained infectious for up to six months in both soils, showing only a 1-log_10_ decline over this period. Decay followed a linear trend, with comparable rates in soil 1 (*k* = 0.215 months^–1^) and soil 2 (*k* = 0.182 months^–1^) (Table S3). The time for a 1-log_10_ reduction (*T*_90_) was 4.6 months in soil 1 and 5.5 months in soil 2, demonstrating a sufficiently long window of persistence for agricultural application. Given the similar phage recovery rates from both soils, the observed decay is likely attributable to gradual phage inactivation rather than immediate adsorption to soil particles. It should be noted that these in vitro experiments were conducted under optimal, dark conditions. In natural settings, environmental factors such as UV radiation, variable moisture, inhibitory components, and host availability would likely accelerate the loss of phage infectivity.

### 3.6 Persistence of ΦNF-1 with Photoprotectants under UV and Sunlight Radiation

To improve the environmental persistence of ΦNF-1, we tested two common agricultural fertilizers, Amino acid 22% (AA22) and Vegepron Sun (CaCO_3_), that can provide photoprotection against UV and sunlight radiation. High-titer phage suspensions were mixed with each photoprotector at 12 and 24% (v/w) and exposed to UV light for 8 h.

At 12%, phage inactivation for both photoprotectors followed a one-phase decay model (Figure 9A, and Table S4). In contrast, at 24%, inactivation shifted to a more stable linear decay. AA22 provided substantially better protection at the higher concentration, reducing the decay rate nearly 3-fold, and increasing the *T*_90_ from 0.33 to 1 h; beyond this point, the infectious phage titer continued to decline gradually over the 8-h period.

**Figure 9:**
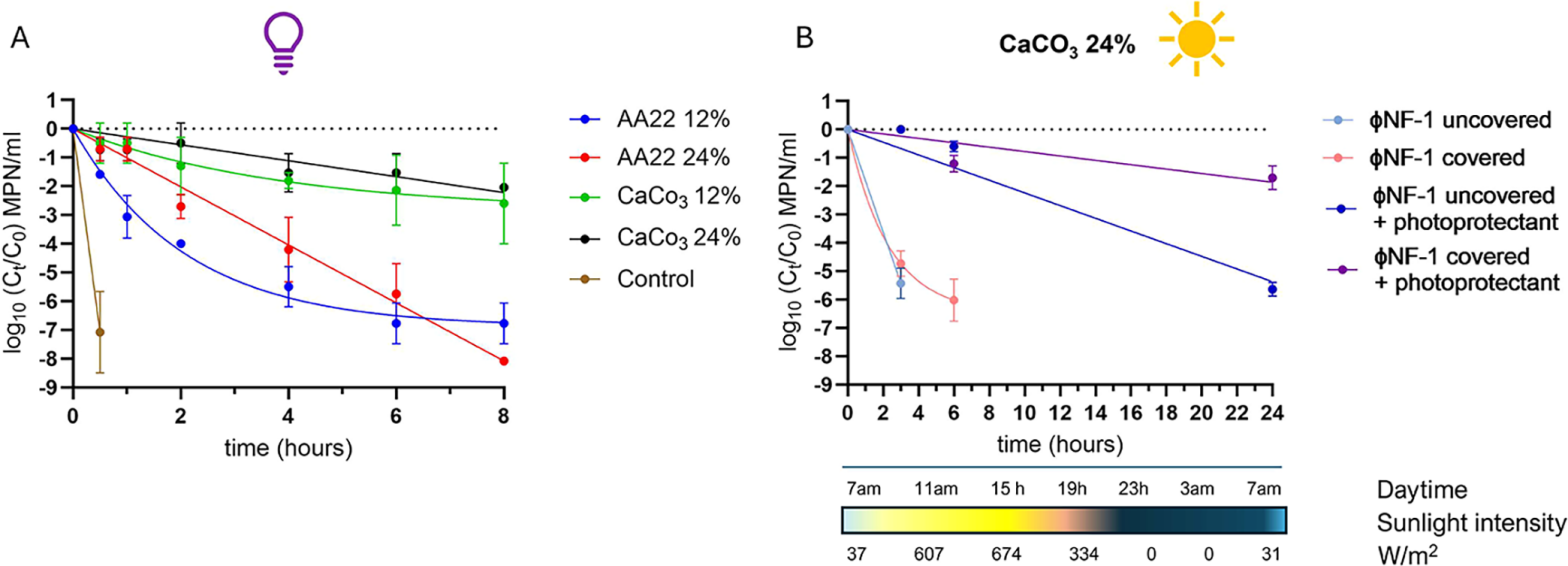
Stability of phage ΦNF-1 under UV and sunlight with photoprotectants. (A) Phage suspensions were mixed with Amino acid 22% (AA22) or Vegepron Sun (CaCO_3_) at 12% or 24% (v/w) and exposed to UV light for 8 h. (B) Phage suspensions with 24% CaCO_3_ were exposed to natural sunlight for 24 h, either in uncovered Petri dishes or covered with lids. Sunlight intensity (W/m^2^) was obtained from the Catalan Forecast Service (*Servei Meterorològic de Catalunya*, *Spain*). The gradient bar indicates daily fluctuations (yellow = highest intensity; dark blue = no sunlight). Error bars indicate standard deviation of three independent experiments.

CaCO_3_ also conferred concentration-dependent protection. At 12%, a 1-log_10_ reduction in infectious phages occurred within 1.6 h, while at 24%, it was delayed to 3.6 h. Decay rates were correspondingly lower at 24% than 12% (*k* = 0.256 h^–1^ vs 0.277 h^–1^), although the final reduction at 8 h was similar for both treatments. When comparing the two photoprotectants at equal concentrations, CaCO_3_ consistently outperformed AA22: the inactivation slopes for AA22 were roughly twice as steep at 12% and five times steeper at 24%.

As a control, phage suspensions in distilled water without photoprotection were rapidly inactivated (*k* = 14.16 h^–1^, *T*_90_ = 0.07 h^–1^) with a complete loss of infectivity within 1 h. These results demonstrate that these agricultural fertilizers can also act as effective photoprotectants, significantly prolonging ΦNF-1 infectivity under UV stress.

Given that 24% CaCO_3_ provided the greatest UV protection, it was further evaluated under natural sunlight for 24 h. Evaporation was a limiting factor due to high outdoor temperatures, so parallel assays were conducted in covered Petri dishes to simulate greenhouse conditions. As in the UV light experiments, CaCO_3_ increased the persistence of infectious phages (Figure 9B). Phage decay followed a linear model (*k* = 0.223 and 0.077 for uncovered and covered plates, respectively). The *T*_90_ values were 5-fold (uncovered plates) and 10-fold (covered plates) higher compared to unprotected controls (Table S4). Without photoprotection, infectious phages fell below the detection limit within 3h (uncovered) or 6h (covered) (Figure 9B). In contrast, phages mixed with 24% CaCO_3_ remained infectious much longer, with *T*_90_ values of 4.8 h (uncovered) and 12.99 h (covered) (Figure 9B and Table S4). These results confirm that CaCO_3_ can effectively shield ΦNF-1 from solar radiation, particularly under covered conditions that protect from UV inactivation and reduce evaporation.

### 3.7 Persistence of ΦNF-1 in Soil under UV and Sunlight Radiation

The persistence of phage ΦNF-1 in soil was assessed in the presence of 24% CaCO_3_, the most effective photoprotectant identified in prior experiments. Sterile agricultural soils were mixed with ΦNF-1 suspensions and exposed to either UV light or natural sunlight. Although soil particles reduced phage persistence compared to suspensions in a culture medium, the effect was not substantial.

Under UV exposure, Petri dish lids effectively blocked radiation, resulting in minimal phage decay (<1-log_10_ reduction over 24 h) (Figure 10A). In uncovered samples, decay followed a segmented linear pattern. The addition of CaCO_3_ enhanced stability during the first 18 h, extending the *T*_90_ from 2.14 h (no photoprotectant) to 9.83 h (Figure 10A and Table S5). Between 18 and 24 h, uncovered samples with CaCO_3_ showed greater decay than those without photoprotection; however, this difference likely reflected high variability among replicates, particularly in the controls.

**Figure 10:**
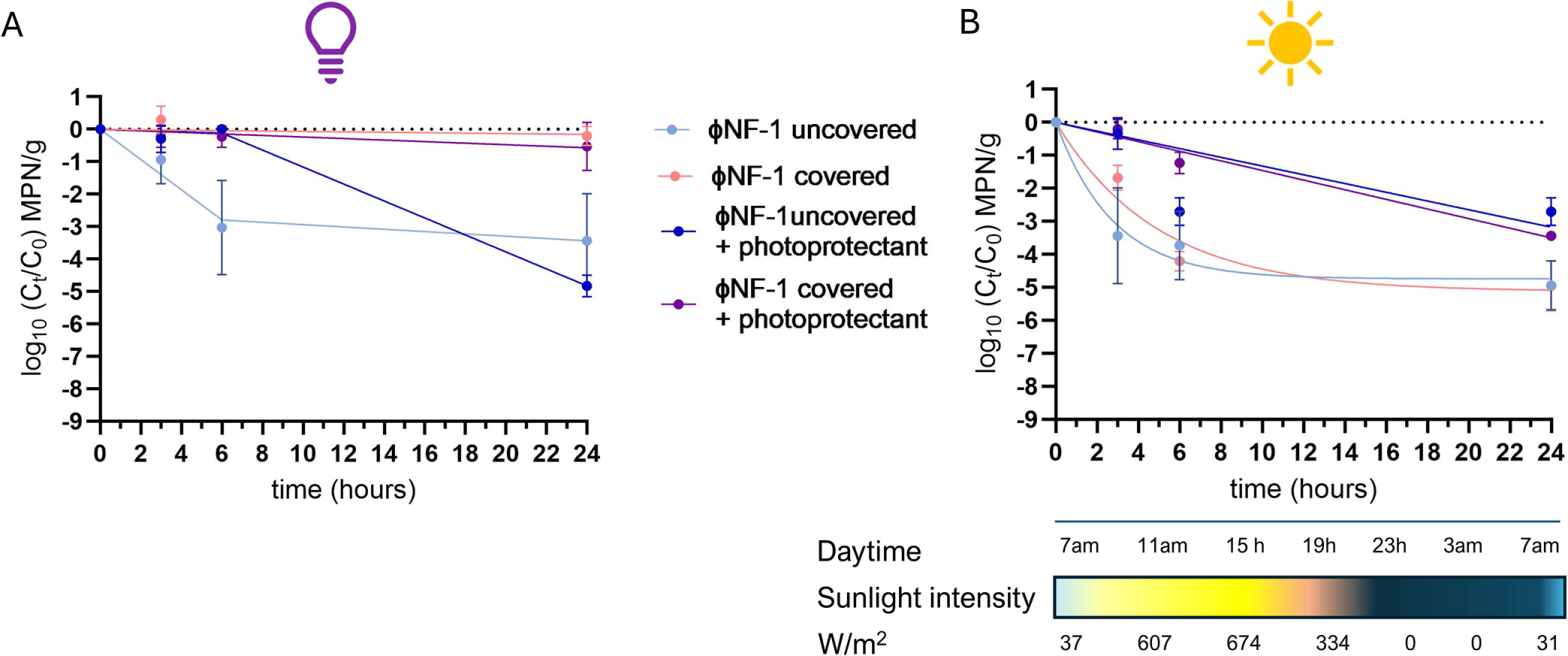
Stability of phage ΦNF-1 in agricultural soil under UV and sunlight with CaCO_3_ protection. Agricultural soils spiked with phage suspension were supplemented with photoprotective agent Vegepron Sun (CaCO_3_) at 24% (v/v) and exposed to UV light (A) or natural sunlight (B) for up to 24 h. Petri dishes were either uncovered or covered with lids to assess differences in protective effects. Sunlight intensity (W/m^2^) was obtained from the Catalan Forecast Service (*Servei Meterorològic de Catalunya*, *Spain*). The gradient bar indicates daily fluctuations (yellow = highest intensity; dark blue = no sunlight). Error bars indicate standard deviation of three independent experiments.

Under natural sunlight (Figure 10B), CaCO_3_ again enhanced phage stability compared to unprotected soils. In CaCO_3_-treated samples, delay followed a linear model, with similar rates under covered (*k* = 0.145 month^–1^; *T*_90_ = 6.9 h) and uncovered (*k* = 0.132; *T*_90_ = 7.6) conditions (Table S5). In contrast, unprotected phages were rapidly inactivated, fitting a one-phase decay model, with *T*_90_ values of only 1.07 h (covered) and 0.60 h (uncovered) (Figure 10, and Table S5).

## 4 Discussion

Bacteriophages targeting AOB represent a promising biological alternative to chemical nitrification inhibitors, with the potential to improve N fertilization efficiency while reducing environmental impacts. Phages infect bacterial hosts, replicate within them, and ultimately cause cell lysis. In recent decades, phages have attracted growing interest as biocontrol agents in diverse fields, including medicine, animal health, plant protection, and food safety.^35–37^

For agricultural applications, the deployment of ΦNF-1 or other AOB-specific phages requires the development of phage-based formulations that can be applied alongside fertilization. Such products must maintain phage infectivity and resilience under field conditions, which necessitates comprehensive evaluation of host range, survival in different types of soil, stability under environmental stressors, and compatibility with additives that may enhance phage activity and longevity.

Among nitrifying microorganisms, *Nitrosomonas* species are the most frequently detected in soils, although other genera, such as *Nitrosospira* and *Nitrosococcus*, also contribute to nitrification.^38^ In addition, ammonia oxidation can be carried out by archaea and to a lesser extent by heterotrophic bacteria and certain fungi.^38,39^ The abundance and distribution of AOB in soils are strongly shaped by environmental factors, particularly soil water content, availability of ammonium, and organic matter content.^40^ The first step of nitrification (the oxidation of ammonia to nitrite by AOB) is the rate-limiting step of the process and a major driver of N loss from agricultural systems, with substantial agronomic and environmental consequences. The application of phage ΦNF-1 has been shown to significantly suppress ammonia consumption during this critical step, thereby reducing nitrification and improving N-use efficiency.^24^ In this study, a multiplicity of infection (MOI) of 0.01 was chosen because it was suitable for our experiments and showed similar performance than higher MOIs (1 and 0.1, Supporting Figure S1), whereas allowed the preparation of larger volumes of phage suspension by diluting the obtained phage stock (average 10^7^–10^8^ MPN) ten- or hundred-fold. The primary objective of this study is to develop an optimized product, and using a lower MOI enables the production of greater quantities from the same batch. Nevertheless, this MOI is not mandatory, and higher MOIs may be more appropriate for field application.

ΦNF-1 has been demonstrated to be a broad-spectrum phage capable of infecting multiple *Nitrosomonas* species (*N. europaea*, *N. nitrosa*, *N. communis* and *N. oligotropha*)^24^ as well as *N. multiformis*. This capacity to target diverse AOB enhances its potential as a biocontrol agent across different soil microbiomes. To our knowledge, ΦNF-1 is the first reported phage capable of infecting more members of the *Nitrosomonas* genera and one *Nitrosospira* species.

Because ΦNF-1 does not form visible lysis plaques, conventional plaque-based assays cannot be used to reliably quantify infectivity. Therefore, we combined multiple complementary approaches to assess infection. Phage propagation was evaluated molecularly through the increase in phage genome copy number, while host cell lysis was inferred from reductions in bacterial genome abundance, decreased nitrite production, and inhibition of pH acidification.

The possibility of a nonproductive “lysis-from-without” effect was ruled out, as phage genome copies increased during infection, and the same phage-to-bacterium ratio did not induce lysis in nonsusceptible strains. Furthermore, electron microscopy supported these findings by demonstrating specific phage attachment to susceptible host cells, whereas no such attachment was observed in nonsusceptible strains.

Our results show that ΦNF-1 remains infectious with minimal losses for up to six months under a range of storage conditions. At neutral to slightly alkaline pH (6–8), phage-containing formulations could be stored for at least six months at 4–20 °C with a ≤1-log_10_ reduction in infectivity, whereas storage at 30 °C led to a slightly greater reduction of 1.2 log_10_ units. In contrast, acidic conditions significantly compromised phage stability, consistent with previous reports for other phages.^41–45^ Acidic environments are known to damage capsid proteins, leading to phage coagulation, precipitation, and loss of infectivity. Additionally, a higher concentration of hydrogen ions may promote phage aggregation, especially when the pH is equal to or below the isoelectric point.^46^ In our experiments, the most plausible explanation for infectivity loss was denaturation of capsid proteins, which impairs host attachment and infection.^47^ Despite the substantial reduction in infectious particles at pH 3 and 5, qPCR showed no decrease in phage DNA, suggesting that capsids remained sufficiently intact to protect the genome, in agreement with previous studies.^48^ The loss of phage infectivity at acidic pH prevents the use of acidic media in phage-based products. Such acidification may occur, for example, when biostimulants based on amino acid hydrolysates are added to the medium, as these hydrolysates are obtained by acid hydrolysis with H_2_SO_4_.^49^ These hydrolysates were not tested in this study precisely because they cause acidification of the medium, and our results showed that this would prevent phage infection. The reduction of phage infectivity under more acidic conditions should also be considered when phages are applied to acidic soils. To evaluate the persistence of infectious phage particles in soil, it is first necessary to determine whether the recovery method can efficiently retrieve phages from the matrix. This is important because phages may adsorb nonspecifically to soil particles and be lost during purification. Our results confirmed that recovery efficiency depends on soil type, with particle size and composition playing key roles. Among the tested matrices, sand showed the lowest recovery, while agricultural soil 1 and garden soil yielded the highest. Poor recovery of phages in sandy soils lacking organic matter has been reported previously.^50^ Although sandy soils are used in agriculture, their low nutrient retention requires higher fertilizer input to maintain crop productivity.^51^ Our findings suggest that phage-based products may be less efficient in sandy soils, mainly due to adsorption to silica (93.2%) alumina (1.3%), and other minerals commonly present in sand such as iron oxides.^50^ For this reason, persistence experiments were carried out in two agricultural soils, which allowed for more consistent recovery and reliable comparisons. In these soils, phage inactivation rates were similar. However, considering that a reduced fraction of infectious phages could not be recovered, the observed 1-log_10_ reduction may underestimate true persistence, as some unrecovered phages likely remained infectious but bound to soil particles.

A key challenge for the field application of a phage-based product is phage sensitivity to environmental stressors, particularly UV radiation, elevated temperatures, and. UV light, whether from a lamp or natural sunlight, compromises phage infectivity by damaging the viral genome directly or indirectly via the generation of reactive radicals. Additionally, UV-induced hydroxyl radicals desiccation can impair viral polymerases, further reducing phage infectivity.^52^

Sunlight inactivation is one of the main challenges for the agricultural application of phages.^53^ One possible strategy is greenhouse cultivation, which is common in vegetable production. Our results suggest that covered conditions improve phage survival, not only by filtering UV radiation but also by preventing evaporation, a major cause of phage inactivation. In addition, we demonstrate that formulation with photoprotectants can markedly increase phage resilience under both covered and uncovered conditions. Although dyes have been proposed as viral photoprotectants,^52^ in this study we selected compounds that have already received EU approval and are commercially available for use in agriculture as biostimulants. As a result, integrating them into phage-based formulations is likely to encounter fewer regulatory obstacles than the introduction of novel substances. Their use therefore constitutes a strategic approach to accelerate regulatory approval and enable the efficient implementation of phage-based biopesticides for the control of bacterial plant pathogens. These additives not only prolong phage persistence but can also promote plant growth. For example, when applied as a foliar fertilizer, CaCO_3_ forms a protective film that improves calcium uptake and provides mechanical protection against abiotic stresses such as sunlight. Beyond photoprotection, this shading effect lowers surface temperature on leaves and fruits, thereby reducing sunburn and heat damage.^54^ In addition, CaCO_3_ fine particles in the phage suspension scatter and reflect UV light, creating an opaque microenvironment.^55^ This lower photon exposure reduce DNA photodamage and the generation of reactive oxygen species that can chemically damage capsid proteins.^56^ In addition, CaCO_3_ solutions retain moisture better because they increase viscosity and create a microfilm that slows evaporation and can maintain alkaline conditions that stabilize phage particles. Together, these effects markedly reduce UV-induced damage to both capsids and viral genomes. In our experiments, CaCO_3_ increased ΦNF-1 persistence under UV radiation, extending survival by up to 7 orders of magnitude compared to untreated controls. While unprotected control suspensions were completely inactivated within 30 min of exposure, CaCO_3_-treated suspensions tolerated UV fluences of 8,553 mJ/cm^2^ after 24 h, with only a 1.5-log_10_ reduction in titer. Under natural sunlight, CaCO_3_ increased persistence by more than 5 log_10_ units in vitro and by 2–3.5 log_10_ units in soil.

Such protection substantially prolongs phage infectivity, which could be useful not only for controlling nitrifying AOB but also for managing phytopathogens in future applications,^19,20^ Based on these findings, incorporation of 24% CaCO_3_ in a phage-based formulation appears highly beneficial for enhancing product stability under field conditions.

This study demonstrates the potential of bacteriophage ΦNF-1 as a sustainable biocontrol agent to reduce nitrification in agricultural soils. ΦNF-1 exhibited an expanded host range, infecting not only *Nitrosomonas* but also *N. multiformis*, which broadens its applicability in diverse soil microbiomes. Persistence assays showed that ΦNF-1 remains infectious for prolonged periods under neutral to alkaline conditions and moderate temperatures, both in aqueous suspension and in agricultural soils, with minimal loss of activity over six months. This high level of stability is compatible with practical storage and field application requirements. The primary challenge identified was phage susceptibility to acidic conditions and solar radiation. However, formulation with 24% CaCO_3_ provided highly effective photoprotection, substantially prolonging phage survival under both controlled UV radiation and natural sunlight.

Phage-mediated suppression of nitrifying bacteria offers a targeted alternative to chemical inhibitors, but its ecological implications must be carefully considered. As with chemical inhibitors, reducing key AOB populations may shift the balance among nitrifiers, enabling compensatory growth of AOA or comammox organisms and altering overall nitrification dynamics. These shifts can cascade through the nitrogen cycle, affecting denitrifiers, nitrate reducers, and heterotrophs that depend on nitrite or nitrate, while changes in ammonium retention may influence competition between microbes and plant roots. Although ΦNF-1 lacks genes associated with lysogeny or transduction, and appears highly specific,^24^ indirect nontarget effects may still arise through altered resource availability or soil chemistry. Moreover, imposing selective pressure on AOB could drive the evolution of phage-resistant strains with modified metabolic traits, potentially reshaping microbial community structure. Collectively, these factors highlight the need for long-term, field-scale studies in different types of soil assessing how phage-based nitrification control influences soil microbial communities and ecosystem functioning.

Overall, our findings provide a foundation for developing ΦNF-1 into a novel phage-based nitrification inhibitor capable of improving nitrogen use efficiency and reducing the environmental impacts of fertilization. This work represents a promising step toward more sustainable agricultural practices.

## Supplementary Material



## Notes

Additional data of infection and stability of ΦNF-1 (Figures S1–S3); and additional data of infectivity and inactivation of ΦNF-1 (Tables S1–S5) (PDF)
