# Supplementary material for: Harnessing the Infectivity and Stability of Bacteriophage ΦNF‑1 in a Phage-Based Product for Sustainable Nitrification Control
Source: Environ Sci Technol. 2026 May 1;60(18):13486–99. doi: 10.1021/acs.est.5c14179 (PMC13173640; doi:10.1021/acs.est.5c14179)
Supplement: Supplementary file 1 [file es5c14179_si_001.pdf]

Supplementary material

Harnessing the infectivity and stability of bacteriophage ΦNF-1 in a phage-based product for sustainable nitrification control

Gloria Vique[1], Pedro Blanco-Picazo[1], Aina Trenchs[1], María Dolores Ramos-Barbero[1], Pablo Quirós[2], Sergio Atares[2], Ignasi Salaet[2], Lorena Rodríguez-Rubio[1], Maite Muniesa[1,3] and Laura Sala-Comorera[1]*.*

*co-corresponding authors. **Maite Muniesa** Phone: +34934039045 e-mail: mmuniesa@ub.edu. **Laura Sala Comorera** Phone: +34934021486 e-mail: laurasala@ub.edu

Summary of contents

Supplementary Figures.- Additional data of infection and stability of ΦNF-1

Figure S1. Pg. S1
Figure S2. Pg. S2
Figure S3. Pg. S3

Supplementary Tables.- Additional data of infectivity and inactivation of ΦNF-1

Table S1. Pg. S4
Table S2.- Pg. S5
Table S3. Pg. S6
Table S4. Pg. S7
Table S5. Pg. S8

Figure S1. Inhibition of nitrification by phage ΦNF-1 in *N. europaea* cultures (10^8 CFU g^{-1}) infected at MOIs of 1, 0.1, 0.01 and 0.01 monitored during 10 days. (A) Inhibition of nitrification was based on NO_2^- accumulation (µM) in the presence or absence (control) of ΦNF-1. (B) Phage propagation (ΦNF-1) of experiments in (A) monitored by qPCR (ΔCt values) in the presence or absence (control) of ΦNF-1. Values represent means of three independent experiments, and errors bars indicate standard deviation. Phage propagation at MOI 0.001 was not detected.

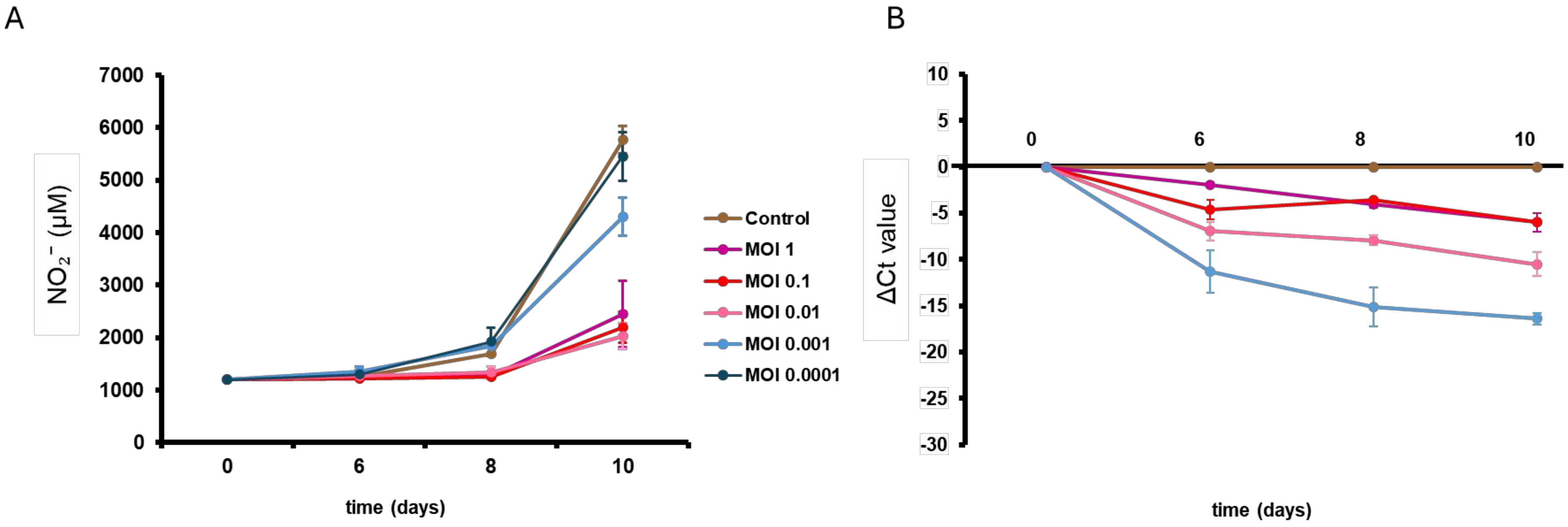

Figure S2. Growth of *N. ureae, N.eutropha.* and *N. briensis* cultures in the presence of phage ΦNF-1 and effects of the phage on ammonium usage. Nitrite concentrations were measured to monitor nitrifying activity (A, C, and E), and pH changes were recorded to determine the growth of bacterial cultures (B, D, and F). Values represent means of three independent experiments, and errors bars indicate standard deviation.

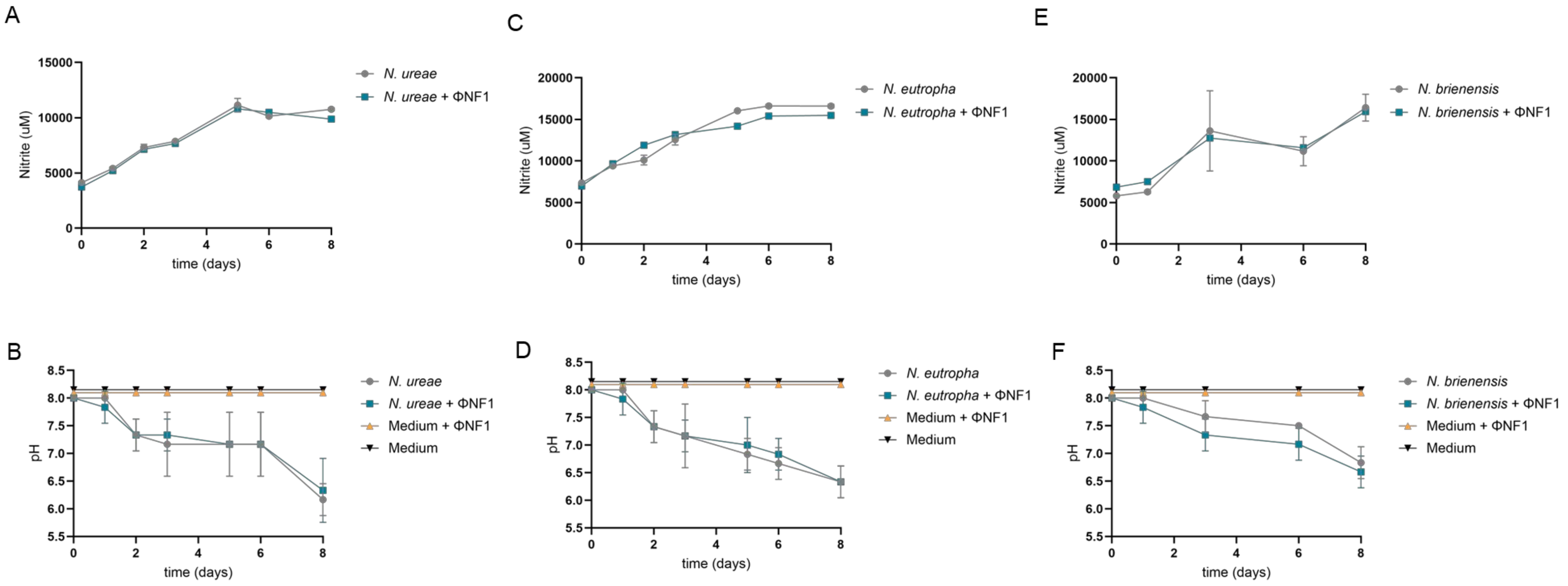

Figure S3. Stability of phage ΦNF-1 DNA under different temperatures and pH conditions. Values are expressed as the difference in Ct between each time point and time 0 of the experiment.

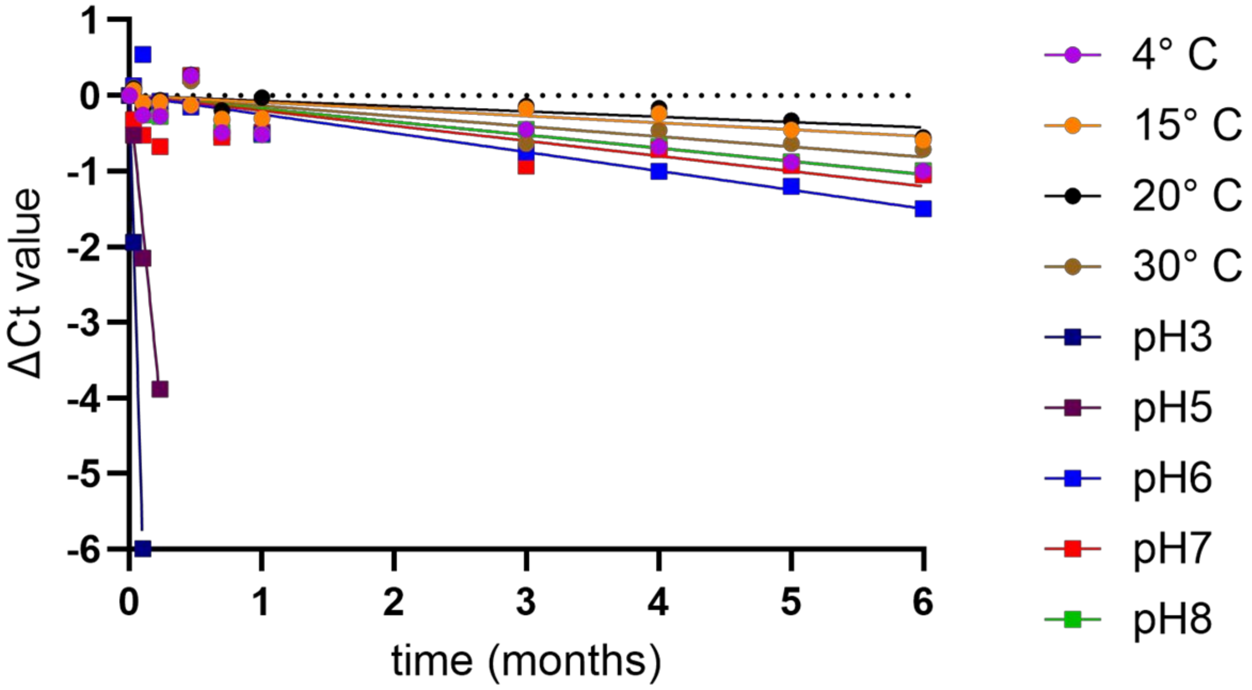

Supplementary Tables

Table S1. Inactivation parameters of phage ΦNF-1 at different temperatures and pHs. The best-fit model (linear regression or one-phase decay) was selected based on the Akaike information criterion (AIC), r^2, and root mean square error (RMSE). k and T_{90} are expressed in $months^{-1}$ and months, respectively.

	Best fitting model	k (m^{-1})<br>(95% CI)	Plateau<br>(95% CI)	r^2	RMSE	Difference in<br>AICc	T_{90} (m)<br>(95% CI)
4 C	Linear regression	0.194<br>(0.142 to 0.246)		0.630	0.226	-2.087	5.15<br>(4.06 to 7.04)
15 C	Linear regression	0.189<br>(0.123 to 0.255)		0.200	0.287	-0.001	5.27<br>(3.92 to 8.13)
20 C	Linear regression	0.175<br>(0.128 to 0.221)		0.613	0.202	-0.201	5.71<br>(4.52 to 7.81)
30 C	Linear regression	0.246<br>(0.191 to 301)		0.696	0.238	-0.012	4.05<br>(3.32 to 5.23)
pH 3	nc						
pH 5	nc						
pH 6	One phase decay	1.973<br>(1.488 to 2.660)	-1.623<br>(-1.488 to -2.66)	0.972	0.109	37.540	0.48<br>(032 to 0.73)
pH 7	Linear regression	0.219<br>(0.151 to 0.286)		0.259	0.292	-0.928	4.56<br>(3.49 to 6.62)
pH 8	Linear regression	0.194<br>(0.142 to 0.246)		0.630	0.226	-2.087	5.15<br>(4.06 to 7.04)

nc: not calculated

Table S2.- Recovery of infectious phage ΦNF-1 in different soil types after 24 h. Initial and 24 h inocula were calculated after propagation in cultures, qPCR evaluation, and MPN analysis as described in the Methods section. Average log_{10} reduction and standard deviation (SD) were calculated from three independent replicates (R1, R2, and R3)

Sample	Replicate	Inital phage inoculum (MPN gr^{-1})	24 h phage titer (MPN gr^{-1})	Log_{10} reduction	Average log_{10} reduction	SD log_{10} reduction
Agricultural soil 1	R1	7.08	6.38	0.70log_{10}		
Agricultural soil 1	R2	7.08	6.04	1.04 log_{10}		
Agricultural soil 1	R3	7.08	6.66	0.42 log_{10}	**0.72**	0.31
Agricultural soil 2	R1	7.08	6.04	1.04 log_{10}		
Agricultural soil 2	R2	7.08	6.04	1.04 log_{10}		
Agricultural soil 2	R3	7.08	5.66	1.42 log_{10}	**1.17**	0.22
Garden soil	R1	7.08	6.38	0.70 log_{10}		
Garden soil	R2	7.08	5.97	1.11 log_{10}		
Garden soil	R3	7.08	6.38	0.70 log_{10}	**0.84**	0.24
Sandy soil	R1	7.08	5.38	1.70 log_{10}		
Sandy soil	R2	7.08	5.48	1.60 log_{10}		
Sandy soil	R3	7.08	5.20	1.88 log_{10}	**1.73**	0.14

Table S3. Inactivation parameters of phage ΦNF-1 in different types of agricultural soils. The best-fit model (linear regression or one-phase decay) was selected based on the Akaike information criterion (AIC), r^2, and root mean square error (RMSE). k and T_{90} are expressed in $months^{-1}$ and months, respectively.

	Best fitting model	k (m^{-1}) (95% CI)	r^2	RMSE	Difference in AICc	T_{90} (m) (95% CI)
Soil 1	Linear regression	0.215 (0.268 to 0.162)	0.686	0.230	-1.53	4.64 (3.72 to 6.15)
Soil 2	Linear regression	0.182 (0.229 to 0.133)	0.685	0.208	-3.38	5.51 (4.36 to 7.48)

Table S4. Inactivation parameters of phage ΦNF-1 in the presence of photoprotective agents: Aminoacid® 22% (AA22) and $CaCO_3$. The best-fit model (linear regression or one-phase decay) was selected based on the Akaike information criterion (AIC), r^2, and root mean square error (RMSE). k and T_{90} are expressed in hours^{-1} and hours, respectively.

		Best fitting model	k (h^{-1}) (95% CI)	Plateau (95% CI)	r^2	RMSE	Difference in AICc	T_{90} (h) (95% CI)
UV	AA22 12%	One phase decay	0.479 (0.340 to 0.659)	-6.891 (-7.784 to -6.207)	0.988	0.284	16.21	0.33 (0.26 to 0.40)
UV	AA22 24%	Linear regression	1.010 (1.087 to 0.932)		0.986	0.348	-6.005	0.99 (0.92 to 1.07)
UV	$CaCO_3$ 12%	One phase decay	0.2567 (0.129 to 0.416)	-2.872 (-4.217 to -2.330)	0.983	0.122	6.918	1.67 (1.34 to 2.09)
UV	$CaCO_3$ 24%	Linear regression	0.277 (0.358 to 0.196)		0.738	0.362	-3.619	3.60 (2.79 to 5.08)
UV	Control	Linear regression	14.16		1			0.07
SL	UC	Linear regression	1.811 (0.142 to 0.246)		1		nc	0.55 (7.04 to 4.07)
SL	C	One phase decay	0.434	-6.497	1		nc	1.07
SL	$CaCO_3$ 24% - UC	Linear regression	0.223 (0.147 to 0.300)		0.952	0.598	nc	4.48 (6.80 to 3.33)
SL	$CaCO_3$ 24% - C	Linear regression	0.077 (0.019 to 0.136)		0.721	0.457	nc	12.99 (52.63 to 7.35)

SL: sunlight, UV, C: covered, UC: uncovered, nc: not calculated

Table S5. Inactivation parameters of phage ΦNF-1 at soils supplemented with $CaCO_3$ as a photoprotective agent. The best-fit model (linear regression, segmented linear, or one-phase decay) was selected using the extra sum-of-squares F test, r^2, and root mean square error (RMSE). k and T_{90} are expressed in $hours^{-1}$ and hours, respectively.

		Best fitting model	k (h^{-1}) (95% CI)	Plateau (95% CI)	r^2	RMSE	F	T_{90} (h) (95% CI)
SL	Soil - UC	One phase decay	0.359 (0.120 to nc)	-4.739 (-3.161 to -6.701)	0.974	0.304	74.69	0.65 (1.97 to nc)
SL	Soil - C	One phase decay	0.201 (0.011 to nc)	-5.126 (-2.644 to -25.01)	0.948	0.518	20.86	1.07 (19.7 to nc)
SL	Soil+ $CaCO_3$ 24% - UC	Linear regression	0.132 (0.012 to 0.277)		0.402	1.137	3.513	7.58 (3.61 to 83.33)
SL	Soil+$CaCO_3$ 24% - C	Linear regression	0.145 (0.141 to 0.177)		0.945	0.248	0.523	6.90 (5.65 to 7.09)
UV	Soil - UC	Segmented linear	k1: 0.466 (0.223 to 0.708) k2: 0.035		0.748	0.869		2.14 (1.41 to 4.48)
UV	Soil - C	not achieved 1 log_{10} reduction						
UV	Soil+ $CaCO_3$ 24% - UC	Segmented linear	k1: 0.019 k2: 0.261 (0.227 to 0.296)		0.988	0.402		9.83 (9.38 to 10.41)
UV	Soil+ $CaCO_3$ 24% - C	not achieved 1 log_{10} reduction						

SL: sunlight, UV, C: covered, UC: uncovered, nc: not calculated
